# Activin A induces ovine follicle stimulating hormone beta using -169/-58 bp of its promoter and a simple TATA box

**DOI:** 10.1186/1477-7827-7-66

**Published:** 2009-06-24

**Authors:** Sang-oh Han, William L Miller

**Affiliations:** 1 Department of Molecular and Structural Biochemistry, North Carolina State University, Raleigh, North Carolina, 27695-7622, USA

## Abstract

**Background:**

Activin A increases production of follicle stimulating hormone (FSH) by inducing transcription of its beta subunit (FSHB). This induction has been studied here in LbetaT2 gonadotropes using transient expression of ovine *FSHBLuc *(-4741 bp of ovine *FSHB *promoter plus exon/intron 1 linked to Luc). Several sequences between -169/-58 bp of the ovine *FSHB *proximal promoter are necessary for induction by activin A in LbetaT2 cells, but deletions between -4741/-752 bp decrease induction > 70% suggesting the existence of other important 5' sequences. Induction disappears if a minimal T81 thymidine kinase promoter replaces the ovine FSHB TATA box and 3' exon/intron. The study reported here was designed to determine if sequences outside -169/-58 bp are important for induction of ovine FSHB by activin A.

**Methods:**

Progressively longer deletions of ovine *FSHBLuc *were created between -4741/-195 bp. Deletions internal to this region were created also, but replaced with substitute DNA. The ovine *FSHB *TATA box region (-40/+3 bp) was replaced by thymidine kinase and rat prolactin minimal promoters, and substitutions were made in 3' intron/exon sequences. All constructs were tested for basal and activin A-induced expression in LbetaT2 cells.

**Results:**

Successive 5' deletions progressively lowered fold-induction by activin A from 9.5 to zero, but progressively increased basal expression. Replacing deletions with substitute DNA showed no changes in basal expression or fold-induction. Induction by activin A was supported by the minimal rat prolactin promoter (TATA box) but not the thymidine kinase promoter (no TATA box). Replacement mutations in the 3' region did not decrease induction by activin A.

**Conclusion:**

The data show that specific ovine *FSHB *sequences 5' to -175 bp or 3' of the transcription start site are not required for induction by activin A. A minimal TATA box promoter supports induction by activin A, but the sequence between the TATA box and transcription start site seems unimportant.

## Background

Follicle stimulating hormone (FSH) is made only in pituitary gonadotropes and stimulates gonads for normal reproductive function in females and males [1-3]. Transcription of the gene encoding FSH beta subunit (*FSHB*) is rate limiting for overall hormone production, and the most potent and influential direct inducer of FSH production is in the activin family [4,5]. Activin A is used to study FSHB regulation in most studies. Significant research has focused on classical Smad activation by activin A and its down-stream signals leading to FSHB expression, but the evidence for Smad involvement with ovine FSHB is not yet clear [6,7].

**Figure 1:**
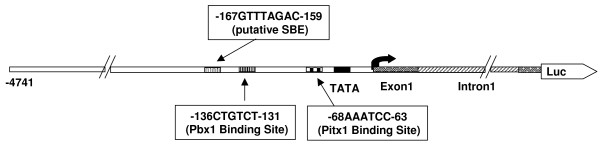
**Diagram of the wild type ovine *FSHBLuc *promoter/reporter construct**. The wild type ovine *FSHBLuc *expression plasmid is shown including -4741 bp of 5' promoter, TATA box (-31/-26 bp), exon 1 (1/63 bp), intron 1 (64/702 bp), part of exon 2 (703/765 bp) and firefly luciferase gene. Regions known to be important for ovine *FSHBLuc *expression are marked: a putative Smad binding site (-163/-159 bp) [7,8], Pbx1 binding site (-136/-131 bp) [8], Pitx1 binding site (-68/-63 bp) [9,10].

A complementary approach to understanding activin A signaling is to identify promoter sequences required for induction. The standard approach for these studies is to analyze transient expression of *FSHB *promoter/reporter gene constructs in transformed murine gonadotropes (LbetaT2 cells). The construct used by our laboratory to study regulation of ovine FSHB is ovine *FSHBLuc *(-4741 bp of ovine *FSHB *promoter plus exon/intron 1 linked to the luciferase gene; see Figure 1).

Transgenic studies recently confirmed that a Smad-related site between -169/-158 bp of the ovine promoter is required for ≥ 99% of ovine FSHB expression *in vivo *[7]. This site was first discovered using transient expression of ovine *FSHBluc *mutants in LbetaT2 cells [8]. More recently transgenic studies were used to confirm the importance of a Pitx1/2 site between -68/-63 bp required for 99% of ovine FSHBLuc expression *in vivo*. This site may have no connection with activin A action in the ovine gene (Sang-oh Han, manuscript in review; our laboratory), but seems to in rodent *FSHB *expression [9,10]. This site is conserved in all mammals studied to date and was first reported to be important using rodent *FSHB*-reporter constructs [9,10]. A third site (Pbx1) is reported to be important for induction by activin A in LbetaT2 cells [8]. Thus, a number of sites in the *FSHB *promoter seem necessary for FSHB expression and, perhaps, regulation *in vivo*.

Interestingly, 5' truncations of rodent *FSHBLuc *constructs are reported to decrease induction by activin A in LbetaT2 cells [11,12]. Truncations from -1990 to -304 bp in mouse constructs reduced fold-induction by 60%. Similar studies with ovine *FSHBLuc *showed that a deletion from -4741 to -750 bp decreased fold-induction by 70% (Pei Su; unpublished results; our laboratory). One interpretation of these data is that there are specific sequences in the 5' region important for activin A action.

Finally, ovine *FSHB *promoter sequences between -4741/-39 bp do not support activin A induction when placed behind the minimal T81 thymidine kinase promoter (Pei Su, unpublished results; our laboratory). By contrast, four copies of the palindromic Smad binding site of the murine *FSHB *promoter do confer activin A induction on a minimal TK promoter/luciferase construct (≥ 10-fold induction) [13]. These results suggest differences between activin A induction of the rodent and ovine genes. One difference could involve the exon and intron that are included in the ovine *FSBHLuc *construct. Alternatively, it could reflect a differential need for a TATA box promoter in the ovine and rodent genes. To date, the 3' ovine intron has been considered important only for basal gene expression by making mRNA processing more efficient and/or effective, but it might also contain sequences needed for activin A induction. Therefore, it is possible that sequences either in the TATA box region or 3' exon/intron region of the ovine *FSHB *gene play an important role in activin A induction.

The study reported here examined the 5', 3' and *TATA *box regions of the ovine *FSHB *gene to determine which regions outside of -169/-58 bp are important for induction by activin A.

## Methods

### Reagents and constructs

Recombinant human activin A was purchased from R&D systems (Minneapolis, MN). FuGENE^®^6 transfection reagent was purchased from Roche Applied Science (Indianapolis, IN) and QuikChange^® ^Site-Directed Mutagenesis kits were obtained from Agilent Technologies Co. (La Jolla, CA). Restriction enzymes including BglII, KpnI, SacI, SacII, Acc65I, AgeI, XhoI, HindIII, and EcoRI as well as dual luciferase assay kits were purchased from Promega (San Luis Obispo, CA). All plasmids were purified using Qiagen midi-prep kits (Qiagen, Valencia, CA). Dulbecco's modified eagle medium (DMEM) and fetal bovine serum were purchased from Invitrogen (Carlsbad, CA). All primers for point mutations were obtained from Sigma-Aldrich Co. (St. Louis, MO). Mutated sections for all plasmids were sequenced by SeqWright Technology (Houston, TX) to confirm sequence correctness, and all common reagents such as yeast extract, tryptone, agar, Tris and boric acid were purchased from Fisher Scientifc Inc. (Pittsburgh, PA). The ovine *FSHBLuc *plasmid used throughout this study (-4741 bp of 5' promoter plus intron 1 linked to a luciferase reporter gene in a pGL3 plasmid) was the same as that reported earlier [14]. Nearly 10 kb of human *FSHB *gene sequence (-3511/+5918 bp) was provided by Dr. T. Rajendra Kumar (University of Kansas Medical Center, Kansas City, KS).

### Transfections in LbetaT2 cells and reporter assays

**Figure 2:**
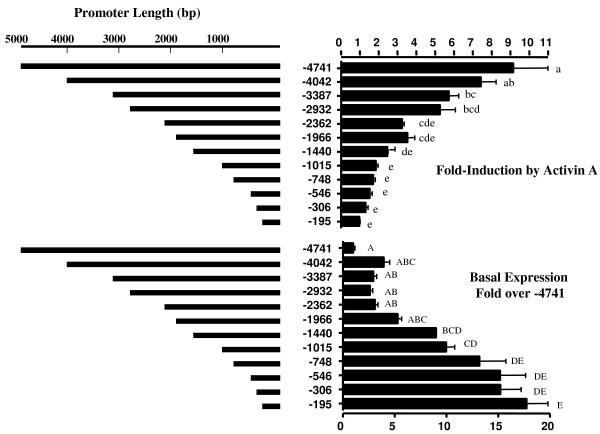
**Deletions: promoter length, fold-induction and relative basal expression**. A series of deletions from -4741 bp to -195 bp produced shorter and shorter lengths of 5' ovine promoter. Promoter length in each construct is plotted proportionately. Opposite each deletion is either the "fold-induction" or basal expression associated with the construct presented as the mean ± sem of quadruplicates in one complete and representative experiment. # designates the bp at the 5' terminus of the deletion. Means with different symbols are statistically different from each other according to analyses by ANOVA followed by Tukey's multiple comparison test using P ≤ 0.05.

**Figure 3:**
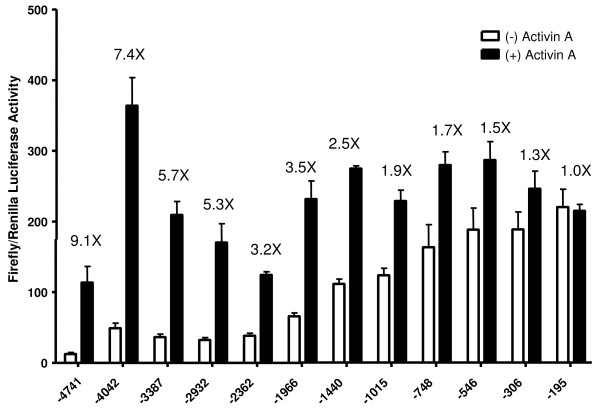
**Deletions: basal expression and induction by activin A**. The raw data used to create Figure 2 are shown. These data were corrected using co-expression of Renilla, but Renilla expression varied ≤ 10% across all samples. Data represent the means ± sem of quadruplicates in one complete and representative experiment. # on the x-axis designates the bp at the 5' terminus of the deletion.

Immortalized murine gonadotropes (LbetaT2 cells) were obtained from Dr. Pamela Mellon (University of California, San Diego, CA) and maintained in complete DMEM containing 10% (v/v) fetal bovine serum plus 100 μg/ml streptomycin and 100 U/ml of penicillin at 37°C under 5% CO_2_: 95% air. For experiments, cells were plated at a density of 30,000 cells in 50 μl of complete media in 96-well tissue culture plates. Cells were transfected 24 hr after plating in quadruplicate by adding 50 μl of serum-free media containing 50 ng plasmid plus 0.15 μl Fugene6. The molecular weights of plasmids with deletions varied significantly (Figures 2 &3) so equal molar amounts of test plasmid were transfected into cells with the weight difference made up by the empty vector.

Cells were co-transfected with 5 ng/well pRL-TK which was used as an internal control since its production of Renilla luciferase was not altered by activin A treatment. After 24 hr of transfection, the media were changed and cells were treated with or without 25 ng/ml activin A for 22 hr prior to harvest with 20 μl of passive lysis solution. Firefly and Renilla luciferase activities were quantified sequentially using a Victor-Light micro plate luminometer (PerkinElmer, Waltham, MA). Assays were performed according to the manufacturer's recommendations. Briefly, 10 μl of cell lysates were pipetted into Costar 96-well half-area microplates (Corning; white; 3693) and 25 μl of firefly luciferase substrate was added for detecting firefly luciferase activity (20 seconds/sample). Luciferase activity was always 40–20,000 × machine background (~400 RLU). After assaying the plate for firefly luciferase, 25 μl of Renilla luciferase substrate plus Stop and Glow was added to specifically detect Renilla luciferase activity. All Renilla activities were 8–100× machine background (~300 RLU) depending on culture preparation. Within individual assays, Renilla activity varied ≤ 10% indicating little variation in transfection efficiency between cultures and/or plasmid preparations. Corrected luciferase activity was calculated by dividing firefly luciferase by Renilla activity and was reported as the ratio of Luciferase/Renilla. Basal activity is defined as activity without activin A treatment. Basal and activin A-induced activities are reported in Figure 3. Fold-induction plotted in Figure 2 was calculated by dividing activin A-induced activity by basal activity shown in Figure 3.

### Deletion constructs in the 5' promoter of ovine FSHBLuc

A series of KpnI restriction sites was created by making point mutations at -4042, -3387, -2362, -1966, -1440, -1015, -546, -195 bp in ovine *FSHBLuc*. Subsequent Kpn1 digestions cut out sequences between these sites and the formerly unique Kpn1 site at -4793 bp of ovine *FSHBLuc*. Re-ligation created 8 of 11 total deletion mutants. Two of the remaining deletions utilized ApaI and EcoRI restriction sites already present in ovine *FSHBLuc *(ApaI at -4786 and -2932 bp; EcoRI at -4744 and -306 bp). The last deletion mutant was made by creating a SacI site at -4775 bp. Subsequent deletion with SacI cut out the fragment between -4775 bp and an existing SacI site at -748 bp. The plasmid was then re-ligated. All mutants were sequenced to verify their structure.

### Replacement constructs in the 5' promoter of ovine FSHBLuc

Point mutations were made to create restriction sites used to excise and replace promoter sequences in ovine *FSHBLuc*. All restriction site mutants were tested to show they had no effect on either expression or activin A-mediated induction of the construct. Replacement 1 (Rep1) was made by replacing -4793/-4043 bp with -1965/-1218 bp using the Kpn1 restriction sites at -4793 and -1965 (see above) and newly made AgeI sites at -4043 and -1218. Replacement 2 (Rep2) was made by replacing -4043/-2760 bp with -2028/-744 bp of ovine *FSHBLuc *using AgeI and BglII restriction sites. Replacement 3 (Rep3) was made by replacing -2760/-2028 bp with +5/+740 bp using BglII and SacII restriction sites. Replacement 4 (Rep4) was constructed by replacing -2028/-744 bp with -4043/-2760 bp after creating AgeI and BglII sites. Replacement 5 (Rep5) was constructed by replacing -750/-175 bp with -3341/-2760 bp after creating SacII and BglII sites. Replacement 6 (Rep6) was constructed by replacing -90/-39 bp of ovine *FSHBLuc *with a synthetic oligonucleotide of the same size in which A and T were exchanged for C and G. The sequence was inserted using AgeI and BglII sites at -90 bp and -39 bp, respectively. Rep6 lacks the Pitx1 site known to be important for rodent and ovine *FSHBLuc *expression and was included as a negative control expected to show low expression and/or low induction by activin A in LbetaT2 cells. Replacement 7 (Rep7) was constructed by replacing sequences at -24/-3 bp, just downstream of the TATA box. The synthetic replacement sequence (random sequence) had a BglII site at the 5' end (see sequence below, lower case) followed by the wild type TATAAA box (bold); an MluI site (lower case) was added at the 3' terminus (-39 agatctTG**TATAAA**CAAGAACAAGAAATGCAacgcgt-3).

### Replacement constructs in 3' ovine FSHBLuc (1 to 701 bp)

Replacement 8 (Rep8) removed exon 1 from ovine *FSHBLuc *(+7/+58 bp) and replaced it with a sequence containing BglII at +5 bp and SacII at +62 bp; the new sequence was synthetically made by substituting original nucleotides as follows: A to C and T to G. Replacement 9 (Rep 9) removed intron 1 from ovine *FSHBLuc *(+83/+660 bp) and replaced it with sequences between -3343 and -2760 bp of the ovine *FSHB *promoter using SacII and BglII sites as done with Rep 5 (see above). This strategy preserved sequences normally associated with splicing such as the GU adjacent to exon 1 and the AG and associated adenosine located several nucleotides upstream of the 5' end of exon 2 [15].

### Thymidine kinase promoter constructs

The p*T109Luc *plasmid, containing 109 bp of the herpes simplex thymidine kinase promoter, was purchased from the American Type Culture Collection (Manassa, VA). Production of ovine *FSHB(TK)Luc *involved substituting the promoter of pT109Luc (-132/+52 bp; named TK in this report) for the same region in ovine *FSHBLuc*. First, AgeI and BglII restiriction sites were created at -40 bp and +3, respectively, in the wild type ovine *FSHBLuc *plasmid. An AgeI restriction site was also created at -132 bp of the pT109Luc plasmid and then sequences between AgeI and an existing BglII site in the pT109Luc promoter were cut and the pT109 promoter (-132/+52 bp) was substituted for wild type sequences in ovine *FSHBLuc*.

### Rat prolactin promoter constructs

Dr. Richard N. Day (University of Virginia, Charlottesville, VA) provided 6X*CRE-37PRL-Luc *which contains 6 copies of a consensus cyclic AMP response element linked to the minimal rat prolactin promoter. Production of ovine *FSHB(rPRL)Luc *involved substituting the minimal promoter of 6X*CRE-37PRL-Luc *(-44/+3 bp; abbreviated r*PRL *in this report) for the same region of ovine *FSHBLuc*. In this case, BglII and AgeI restriction sites were created at -39 bp and +3 bp, respectively, in ovine *FSHBLuc *and BglII and AgeI sites were created at -44 bp and +3 bp, respectively, in 6X*CRE-37PRL-Luc*. This allowed substitution of the minimal rat prolactin promoter for the equivalent wild type promoter sequence in ovine *FSHBLuc*. Production of ovine Δ-175/-39 *FSHB(rPRL)Luc *involved creating a BglII site at -175 bp of ovine *FSHB(rPRL)Luc *followed by digestion with BglII which excised sequences between -175 and -39 bp. The construct was re-ligated to produce ovine Δ-175/-39 *FSHB(rPRL)Luc*. Replacement 10 (Rep10) was constructed from ovine *FSHB(rPRL*) by replacing sequences between KpnI (-4793 bp) and EcoRI (-207 bp) with human coding sequence between +1049/+5939 bp. An EcoRI site was put into ovine *FSHB(rPRL) *at -207 bp and the large segment between KpnI (-4793 bp) and EcoRI was excised from the construct. The human sequence was obtained from human *FSHB*-GEM3 after creating an EcoRI site at +5939 and using an existing ACC651 site at +1049 bp (compatible with KpnI) of human *FSHB*-GEM3. These sites were cut with Acc65I and EcoRI and the resulting fragment was ligated into ovine *FSHB(rPRL) *to produce Rep 10. All constructs were sequenced for verification of structure.

### Statistics

**Figure 4:**
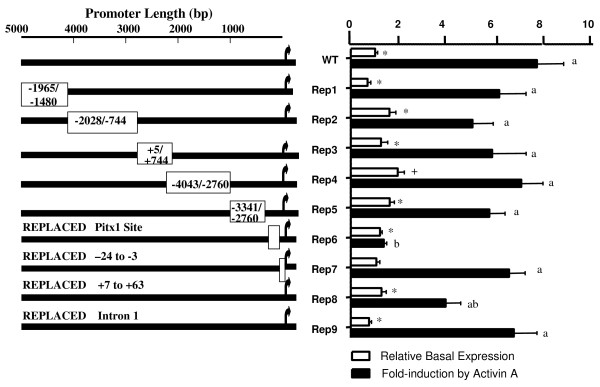
**Replacement mutations between -4741/+701 bp of ovine *FSHBLuc***. Ovine *FSHBLuc *was divided into 9 sections to determine where activin-responsive sequences might be located. For replacement constructs 1–5, deleted regions are designated with a box that indicates what sequences were substituted for the deletion. For replacement constructs 6–9, the section deleted and replaced is written above the promoter line. The replacement sequences for 6–9 are identified in Materials and Methods. Fold-induction was calculated as a ratio of luciferase expression ± activin A corrected for Renilla co-expression. This Luciferase/Renilla ratio was 54 for WT induction. Basal expression was calculated the same way and normalized to wild type basal expression (WT = 1). The ratio of Luciferase/Renilla was 10 for WT basal expression. Bars represent the mean ± sem of results from quadruplicate cultures from a single but representative complete experiment. Means with different letters are statistically different from wild type induction (fold-induction). Means with different symbols are statistically different from wild type basal expression (relative basal expression). ANOVA and Student Newman-Keuls analyses were used to determine significance between means using P ≤ 0.05.

**Figure 5:**
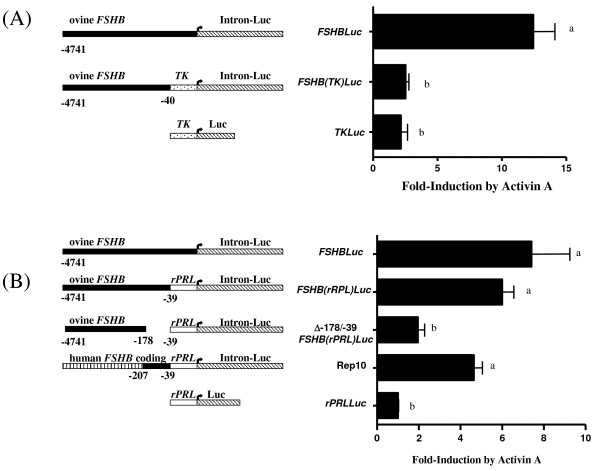
**Activin A induction of ovine *FSHBLuc *with minimal promoters**. (A) Induction of wild type ovine *FSHBLuc *by activin A was compared to induction of ovine *FSHB(TK)Luc *with a substitute thymidine kinase promoter (TK) placed between -40/+3 bp of ovine *FSHBLuc*. Induction of the parent TK construct is also shown. (B) Induction of wild type ovine *FSHBLuc *by activin A is compared to induction of ovine *FSHB(rPRL)Luc *with a substitute rat prolactin minimal promoter placed between -40/+3 bp of ovine *FSHBLuc*. Induction by these constructs was then compared to induction of the rat promoter construct lacking sequences known to be important for activin A induction Δ-175/-39 ovine *FSHB(rPRL)Luc*. The next construct contained the minimal rat prolactin promoter plus ovine 3'exon/intron 1, but lacked all 5' ovine sequence except the region between -200/-39 bp known to be important for activin A action (Rep 10). Finally, induction with activin A was measured for the original rat prolactin construct. The data in Figure 5 are plotted as fold-induction since basal expression with the *TK *and *rPRL *promoters was increased 20- and 8-fold, respectively, compared to basal expression with the wild type promoter. Bars represent the mean ± sem of results from quadruplicate cultures from a single and representative experiment. Means with different letters were statistically different from wild type induction (fold-induction). ANOVA and Tukey's multiple comparison analyses were used to determine significance where P ≤ 0.05.

All experiments were repeated 3 times or more with similar results. LbetaT2 cells respond with significant variation to activin A over time, so pooling results from different experiments makes it very difficult if not impossible to show statistical differences from different constructs or treatments. Therefore, results are plotted as representative data from one complete experiment performed with quadruplicate cultures. For Figures 2, 3 and 5A/B, statistical comparisons between multiple samples were accomplished using ANOVA followed by Tukey's multiple comparison test. For Figure 4, statistical comparisons between multiple samples were performed using ANOVA followed by Student Newman-Kuels analysis. Statistical calculations were performed using Prism version 4 (GraphPad software, Inc., San Diego, CA)

## Results and discussion

### Ovine FSHB promoter sequences between -4741/-175 bp were not required for activin A induction

Important 5' promoter sequences between -169/-158 bp and -68/-63 bp were previously identified by a number of researchers using small targeted deletions in conserved regions of rodent or ovine *FSHB *promoters. Their identification did not involve large scale 5' deletions, and the importance of these specifically targeted regions has been documented in vitro and partly in vivo as outlined in the Background section.

Deletion studies with the 5' *FSHB *promoters of mouse [11] or ovine (Pei Su; unpublished results; our laboratory) also revealed major changes in fold-induction by activin A. Studies in 2001 showed that a deletion between -4741/-750 bp in the 5' promoter of ovine *FSHBLuc *(Figure 1) decreased activin A induction of this construct by 70% in LbetaT2 cells. This deletion also increased basal expression 14×. These results suggested that an important silencer might be in this region. This study was initiated to explore this possibility. As a result, many sequential deletions were made between -4741/-195 bp. All these deletions were upstream of the known response elements between -169/-58 bp.

The deletion data in Figure 2 indicate that "fold-induction" progressively decreased from 9.5-fold to zero induction as promoter length shortened. This was considered odd because we already knew that sequences downstream of -195 bp were important, but the data suggested either that important 5' sequences existed upstream of -195 bp or that plasmid artifacts had been generated. Plotting promoter length versus "fold-induction" highlighted the direct progressive correlation between promoter length and fold-induction (Figure 2). No single deletion or even pair of deletions caused a significant decrease in fold-induction although fold-induction steadily decreased with apparent loss of activin A effectiveness. As noted in 2001 (Pei Su), an opposite effect was observed for basal expression which increased as promoter size decreased (Figure 2). A significant increase occurred when sequences between -1966/-748 bp were deleted suggesting that a silencer might reside in this 1218 bp region, but no significant change occurred in basal expression when sequences between -4042/-2362 were deleted indicating there were no potential silencers in this more distal region.

Figure 3 depicts the raw data used to generate Figure 2. In fact, all data in Figure 2 can be calculated from Figure 3, but Figure 3 does not show the tight relationships that exist between fold-induction, basal expression and promoter length. Figure 3 does show, however, that activin A-induced expression was relatively constant for all constructs compared to basal expression. Shorter constructs (-1996 to -195) did express slightly better with activin A treatment, however, than the longer constructs.

Based on the data in Figures 2 and 3, it could be postulated that deletions eliminated a silencer between -1966/-748 bp. If this were so, deletion relieved tonic repression of *FSHBLuc *and the need/ability of activin A to overcome it. The region between -1966/-748 bp would then be very important for regulation of FSHB by activin A *in vivo*. However, the gentle decline in "fold-induction" and increase in basal expression also suggested a non-specific effect.

To test these two hypotheses, 5 deletions were created and replaced with substitute sequences of equivalent length DNA to preserve original spacing in the plasmid (Figure 4, see WT versus replacements 1–5). Replacements 2 and 4 (Figure 4) were made specifically to test the hypothesis that a silencer might reside between -1966/-748 bp since basal expression increased significantly when this section was deleted. Replacement 2 contained two copies of this putative "silencer" region. By contrast, Replacement 4 entirely lacked the putative "silencer" region and contained two copies of sequences between -4043 and -2760 which showed no effect on basal expression when they were deleted. The results shown in Figure 4 indicate that both "fold-induction" and basal expression for these two replacement constructs were statistically identical when compared to each other by ANOVA and Student Newman-Kuels analyses. Therefore, it was concluded that a silencer does not exist in the -1966/-748 bp region. Likewise, sequences between -4043/-2760 contained no special sequences associated with activin A induction.

Other replacement DNA sequences came from a coding sequence or other parts of the ovine *FSHB *promoter, but all of these constructs had similar basal and activin A-induced expression. Because major changes in fold-induction by activin A disappeared when construct size was maintained, it was concluded that changes in fold-induction due to progressive 5' deletions was an artifact caused by some non-specific effect (see discussion at the end of this section).

The above results and discussion about the distal ovine promoter are not meant to imply that 5' sequences have no relevance for FSHB expression. Transgenic studies with full length and truncated ovine *FSHBLuc *constructs indicate that 5' sequences somewhere between -2100 bp and -750 bp are needed for pituitary expression *in vivo *[14]. Kato *et al *also reported that Prop 1 binding sites between -852/-746 bp in the porcine *FSHB *promoter are required for expression *in vivo *[16,17], and the ovine *FSHB *promoter shares high homology with this porcine promoter region. The data in Figures 2, 3, 4 of this report were focused on activin A induction of ovine *FSHBLuc *in LbetaT2 cells, but it appears that LbetaT2 cells cannot detect all sequences required for gonadotrope expression in vivo.

Interestingly, other investigators have found results that are almost identical to those observed by us using progressive deletions, but with different gene promoters. These investigators also concluded that 5' silencers must exist which means they will also design experiments to find silencers. One research group also used a pGL3 plasmid (like ours) for investigating expression of the rodent selenoprotein W [18]. The other study used a different plasmid (pNAGM) to study regulation of heat shock protein 30 in *Aspergillus Oryzae *[19]. The latter report contained a figure similar to Figure 2 of this report. Both research groups may find silencers, but they may also be witnessing a plasmid-made artifact as in our case.

It is unclear why this phenomenon exists in our ovine *FSHBLuc *constructs. It may involve bringing backbone plasmid sequences closer to the eukaryotic transcription start site enabling a cryptic enhancer to increase expression until it overshadows induction by activin. Care has been taken, however, to eliminate such effects by companies that sell expression plasmids. There might also be an effect caused by the packaging of closed circular DNA into nucleosomal structures [20]. The mechanism is unclear, but the phenomenon is real.

### Ovine FSHB 3' sequences (exon/intron 1) were not needed for induction by activin A

Figure 4 also shows that replacement of the first 3' exon (Rep 8) and intron 1 (Rep 9) had little or no effect on activin A induction. Replacing intron 1 with neutral sequence from the 5' promoter caused no difference in basal or activin A-inducted expression compared to WT expression. Likewise, replacing exon 1 with a synthetic oligonucleotide (switching A/C and T/G) caused no significant difference between induction by activin A compared to WT induction. Based on these data, it was concluded that 3' sequences in the first exon and intron (+7/+701 bp) were not required for activin A induction of ovine *FSHBLuc*.

### The importance of a minimal TATA box promoter

Figure 4 (Rep 7) shows that synthetically changing the 22 bp between the ovine TATA box and transcription start site of wild type ovine *FSHBLuc *did not alter activin A induction; there is no homology between wild type and synthetic sequences (aggtgaactgagactagactcagc was changed to caagaacaagaaatgcaacgcgtc). However, substitution of the TATA box region with the entire minimal TK promoter (TK109) did destroy activin A induction (Figure 5A) as observed previously when the T81 promoter was used. It may be that this promoter with its GC and CAAT boxes increased basal expression so high (20-fold higher than WT) that activin A action was overshadowed by basal expression much like that observed with 5' deletions. Nevertheless, the 20 bp between the TATA box and transcription start site of rat prolactin (agtcaatgtctgcagatgag; no homology to either ovine or synthetic sequences in Figure 4, Rep 7) also increased basal expression (8×) but fold induction by activin A was fully preserved (Figure 5B). We conclude that the TATA box is important, but that the sequence between the TATA box and transcription start site is unimportant.

The data in Figure 5B show that the minimal rat prolactin promoter (with TATA box) did support maximal activin A induction when the full length ovine *FSHB *promoter was attached to it. When the distal 5' region was replaced with apparently neutral sequences from the 3' human *FSHB *coding gene, the rat promoter was still able to support activin A induction as long as the construct contained ovine sequences between -207/-39 bp. Deletion of the ovine promoter between -175/-39 bp did destroy activin A induction, as predicted, since these sequences are necessary for activin A induction of wild type *FSHBLuc *expression in LbetaT2 cells [7-10]. Therefore, it was concluded that the -169/-58 bp segment which is known to be essential for activin A induction of ovine FSHB promoter plus a minimal TATA box comprise all necessary and sufficient sequences required for activin A induction of ovine FSHB expression, at least, in LbetaT2 cells.

### Insights from transgenic studies

Both human and ovine *FSHB *genes have been successfully expressed as transgenes in mice and both are regulated as if they were endogenous mouse *FSHB *genes [1,14]. The shortest 5' sequence tested with the human gene contained -350 bp of 5' sequence. This includes the region noted in this report (-169/-58 bp) along with the human TATA box region. Clearly, there are other important sequences in the human and ovine genes important for gonadotrope expression as well. In the human gene these sequences reside between + 3142 and +2138 in the 3' region of the gene [1]. As noted above, the ovine *FSHB *gene needs sequences upstream of -750 bp [14], but both human and ovine genes require proximal promoter sequences within -300 bp of the transcription start site for proper expression in vivo, which is consistent with the data presented in this paper.

It will ultimately be important to confirm the conclusions of the studies reported here in transgenic mice using artificial gene promoters containing only those sequences thought important for gene expression and regulation. As noted above for the ovine *FSHB *gene, there is still one promoter element upstream of -750 bp that remains undefined but necessary for gonadotrope expression [14]. This site must be defined before a synthetic promoter with defined elements can be tested as a transgene in vivo. LbetaT2 cells appear insensitive to this element so we are currently investigating this site using viral constructs and primary gonadotropes [2].

## Conclusion

The combined data reported in this and previous studies showed that the critical sequences for activin A induction of ovine *FSHBLuc *are located between -169/-58 bp plus the simple generic TATA box found in ovine *FSHBLuc *or the rat prolactin promoter. Neither the distal 5' region (-4741/-175 bp) nor 3' exon/intron sequences were found important for activin A induction of ovine *FSHBLuc *in LbetaT2 cells. The necessary and sufficient sequences for activin A-inducible ovine FSHB expression comprise sequences only between -169/-58 bp plus a minimal TATA box promoter.

## Competing interests

The authors declare that they have no competing interests.

## Authors' contributions

SH participated in the design of the study, performed all DNA mutations, transfected the constructs in LbetaT2 cells, performed luciferase assays, analyzed the data, and wrote the manuscript. WLM participated in the design of the study, helped interpret the data, and revised the manuscript. SH and WLM read and approved the final manuscript.
